# Melittin as a therapeutic agent for rheumatoid arthritis: mechanistic insights, advanced delivery systems, and future perspectives

**DOI:** 10.3389/fimmu.2024.1510693

**Published:** 2024-12-20

**Authors:** Ashutosh Pareek, Khushbu Mehlawat, Kritika Tripathi, Aaushi Pareek, Simran Chaudhary, Yashumati Ratan, Vasso Apostolopoulos, Anil Chuturgoon

**Affiliations:** ^1^ Department of Pharmacy, Banasthali Vidyapith, Banasthali, India; ^2^ School of Health and Biomedical Sciences, RMIT University, Melbourne, VIC, Australia; ^3^ Discipline of Medical Biochemistry, School of Laboratory Medicine and Medical Sciences, University of KwaZulu-Natal, Durban, South Africa

**Keywords:** rheumatoid arthritis, bee venom, melittin, anti-inflammatory, novel formulations

## Abstract

Rheumatoid arthritis (RA), a condition characterized by joint deterioration through the action of matrix metalloproteinases (MMPs), is prevalent worldwide. Bee venom (BV) has traditionally been used in Chinese medicine for pain, arthritis, rheumatism, skin diseases, etc. BV is enriched with active substances, notably melittin and phospholipase A2 (PLA2), offering significant therapeutic potential. Hence, the review summarizes current insights into BV’s composition, antiarthritic mechanism and pharmacological benefits, focusing on melittin. Constituting 50-60% of BV, melittin notably downregulates nuclear factor Kappa B (NF-κB) activity, inhibits MMP-1 and MMP-8, and diminishes tumor necrosis factor (TNF-α), all of which contribute to the mitigation of type 2 collagen degradation. Despite its potential, melittin exhibits hemolytic activity and can significantly affect cell membranes, limiting its application, which poses a challenge to its therapeutic use. To overcome these challenges, delivery techniques utilizing nanocarriers and modifications in amino acid sequencing have been developed. Recent advancements in delivery systems, including nanocarriers, transdermal patches, and nanoemulsions, aim to minimize toxicity, expanding its therapeutic utility for RA. This article explores these novel strategies, underlining the evolving role of melittin in RA management.

## 1 Introduction

Rheumatoid arthritis (RA) is a chronic autoimmune inflammatory disease affecting up to 1% of the population in developed countries. Environmental factors, genetic predisposition, and smoking are significant contributors to the development of RA (1). RA prevalence is higher in industrialized regions, likely due to demographic factors. According to the WHO, in 2019, RA impacted around 18 million people globally, with 70% of cases occurring in women and 55% in individuals over the age of 55 (2). Of those affected, 13 million experienced moderate to severe symptoms and benefited from rehabilitation efforts (3).

In recent decades, the incidence of RA has increased significantly, and this trend is expected to continue (4). Research highlights a considerably higher prevalence of RA in women compared to men (5). The etiology of RA is complex, involving interactions between environmental factors, the microbiome, mucosal health, and host immune function (6). Typically, the disease initiates in the mucosal tissues, where various inflammatory cytokines, immune cells, and signaling pathways become involved. This process eventually leads to interactions between the mucosal immune system and dysregulated microbiota, which then migrate to the synovium and joints (1, 7).

**Figure 1:**
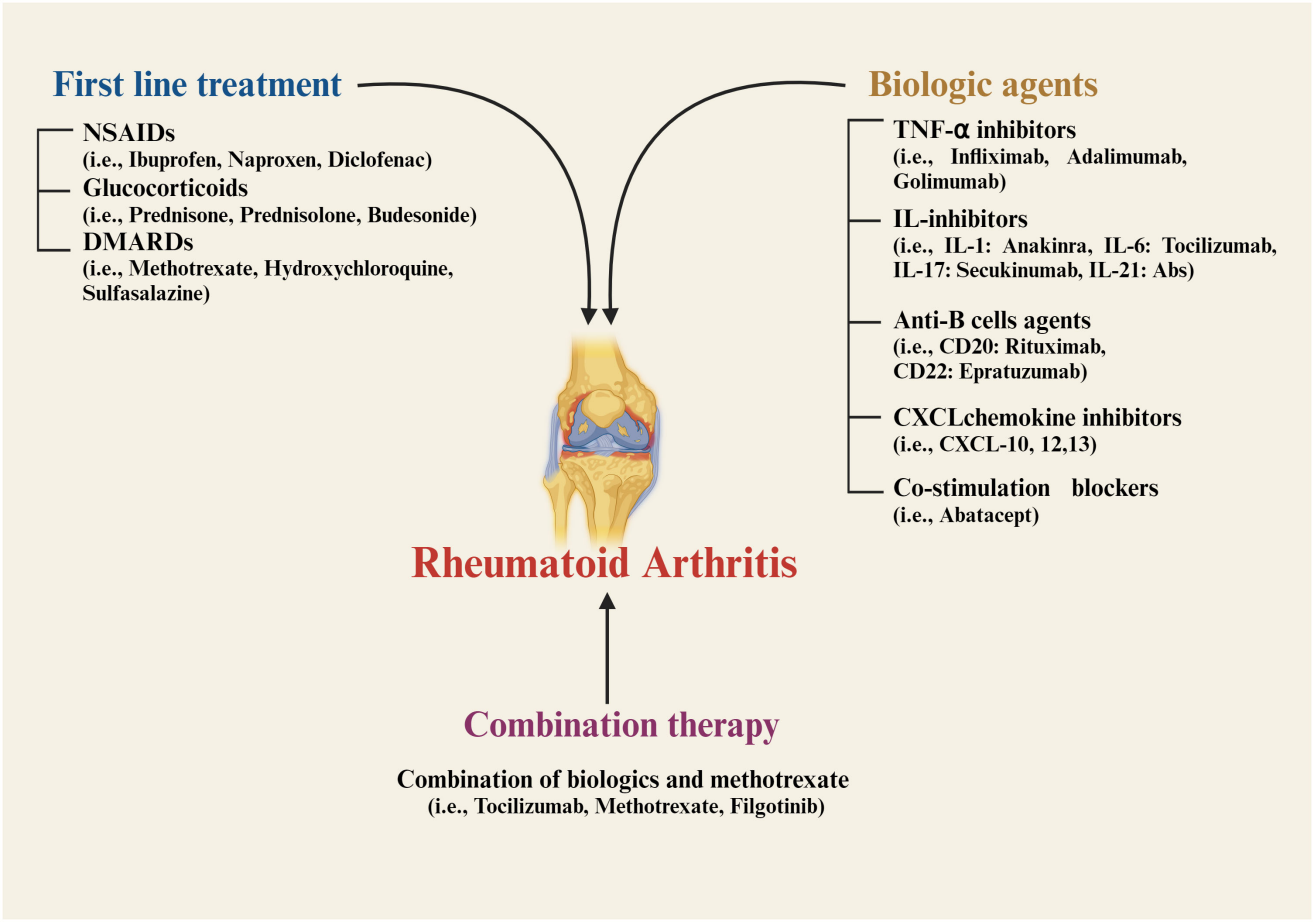
Current strategies for treatment of Rheumatoid arthritis. NSAIDs, nonsteroidal anti-inflammatory drugs; DMARDs, disease-modifying anti-rheumatic drug therapy; TNF, tumor necrosis factor; IL, interleukin; CXCL, CXC chemokine ligand; CD, cluster of differentiation.

This chronic condition is characterized by inflammation of the synovial membrane, leading to joint damage, swelling, stiffness, pain, tissue degradation, deformity, and instability. The disease usually progresses symmetrically (8–10). Key factors driving inflammation in RA include tumor necrosis factor (TNF-α), interleukin-6 (IL-6), and other cytokines (10, 11). The immune response in RA is triggered by cytokines and chemokines, which further activate B and T-cells, macrophages, and monocytes, leading to excessive swelling (12, 13). Currently, nonsteroidal anti-inflammatory drugs (NSAIDs) (13), glucocorticoids (14), and disease-modifying anti-rheumatic drug therapy (DMARDs) (8, 10, 15) are the first line of treatment for RA therapeutics (**Figure 1**). However, NSAIDs may lead to gastrointestinal bleeding and other adverse effects, while DMARDs can cause immune suppression, increasing the risk of infections (12, 16, 17). Recent advancements in RA therapy have introduced biologic agents targeting TNF-α and interleukins (ILs) (18–21). These biologics inhibit specific immune components, such as CXC chemokine ligand inhibitors (22, 23), anti-B-cell agents (24–26), and T-cell co-stimulation blockers (27). Combination therapies that include biologics and methotrexate are also commonly used (28–30). Additionally, synthetic agents targeting Janus-activated kinase (JAK) inhibitors and cell therapies utilizing mesenchymal stem cells (MSCs) have been incorporated into RA treatment protocols (29–31). Biologics, although targeted and effective, are often expensive, making them inaccessible for many patients (17, 32).

Despite these treatment advancements, many RA patients face significant side effects and limited efficacy from existing therapies, leading to suboptimal treatment outcomes and continued discomfort (32, 33). In response to these challenges, researchers are exploring alternative therapies, including medicinal plants and animal-derived drugs, to provide more effective relief from this autoimmune disorder (17, 34–39). One of the traditionally used animal-derived therapies is bee venom (BV), which has been used in China for centuries to treat inflammation and pain, a practice known as ‘Apitherapy’ (40, 41). Apitherapy involves the use of bee products for the treatment or prevention of disease. BV is particularly noted for its anti-inflammatory properties, primarily due to its main active component, melittin (42). The therapeutic efficacy of BV in managing rheumatism and arthritis is thought to begin with the activation of adrenal glands that produce cortisol. Recent studies have documented various pathways through which BV and its components exert anti-inflammatory or antiarthritic effects (43, 44). Melittin, a small protein consisting of 26 amino acid residues, appears to downregulate phospholipase A2 (PLA2), cyclooxygenase (COX-2), and TNF-α expression while reducing levels of IL-1β, IL-6, nitric oxide (NO), and reactive oxygen species (ROS) (45).

## 2 Pathophysiology of rheumatoid arthritis

**Figure 2:**
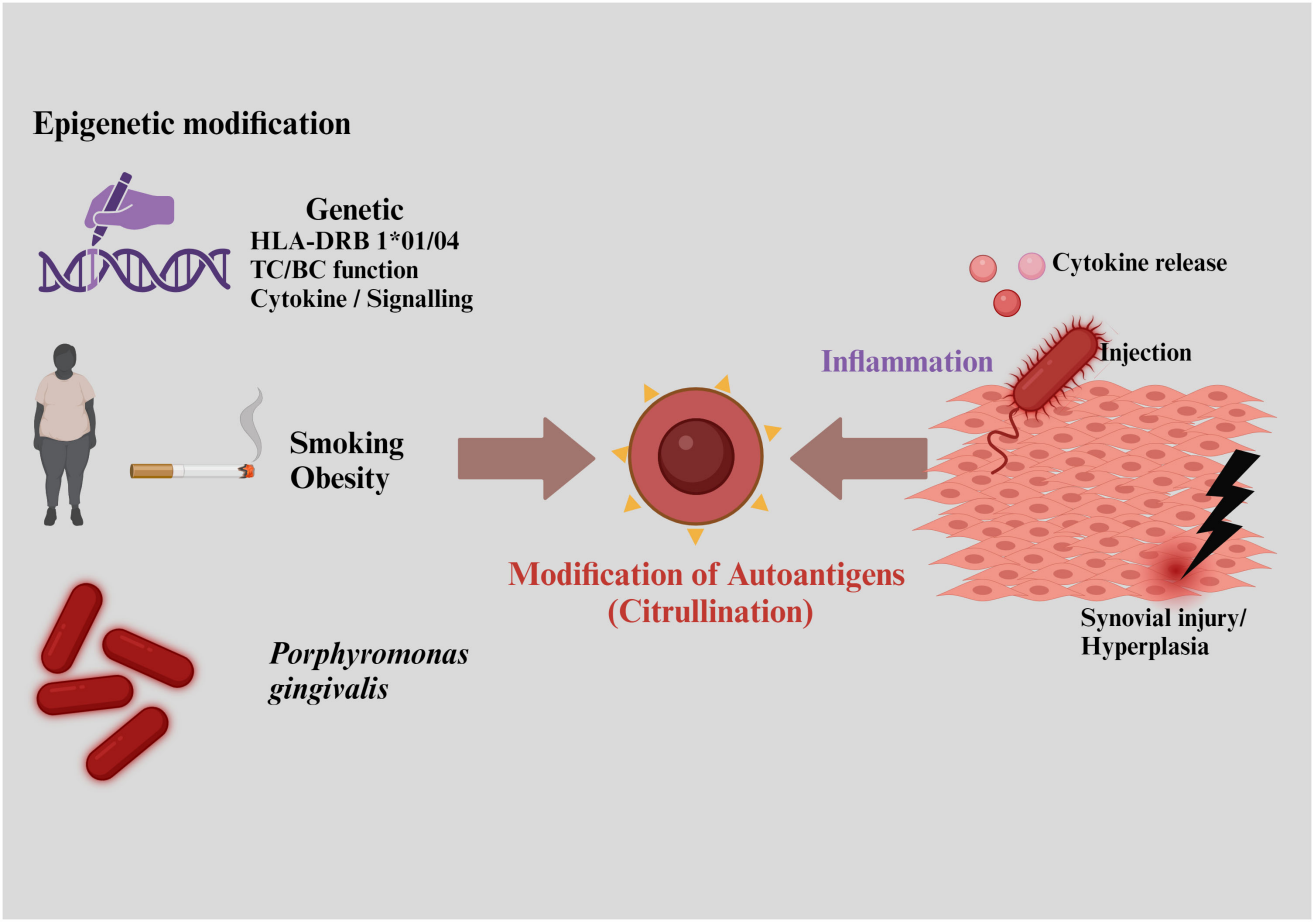
Risk factors responsible for rheumatoid arthritis include interactions with environmental factors, smoking, and genetic predispositions, leading to the production of autoantibodies against citrullinated antigens.

RA is primarily characterized by hyperplasia and inflammation of the synovium, leading to progressive destruction of cartilage and bone. This condition is associated with a range of systemic complications, including cardiovascular, pulmonary, and psychological disorders (46). RA typically stems from a breakdown in immune tolerance, producing a symmetric pattern of synovial inflammation. This breakdown often results from complex interactions between environmental factors, smoking, and genetic predispositions, triggering the production of autoantibodies against citrullinated antigens—a hallmark of RA (**Figure 2**) (47, 48).

RA is classified into two major subtypes based on the presence or absence of anti-citrullinated protein antibodies (ACPAs), detectable in around 67% of patients and serving as a key diagnostic marker (49). ACPAs production is typically triggered by environmental factors and epigenetic changes, which often involve a combination of genetic and environmental influences (47). Likely trigger sites for RA include the lungs and gut, where interactions initiate autoantibody production against citrullinated peptides, marking the onset of self-protein citrullination. Lung exposure to infectious agents like *Porphyromonas*, *Epstein-Barr* virus, and gingivitis, along with noxious agents, dietary factors, and the gut microbiome, also contribute to ACPA generation (50).

Citrullination is catalyzed by the calcium-dependent enzyme protein arginine deaminases (PAD), converting neutral arginine into a polar citrulline residues in granulocytes and macrophages in RA patients (50). An abnormal antibody response produces ACPAs targeting various citrullinated proteins, including histones, type 2 collagen, Epstein-Barr nuclear antigen 1, α-enolase, vimentin, and fibronectin, contributing to the systemic nature of RA (50).

**Figure 3:**
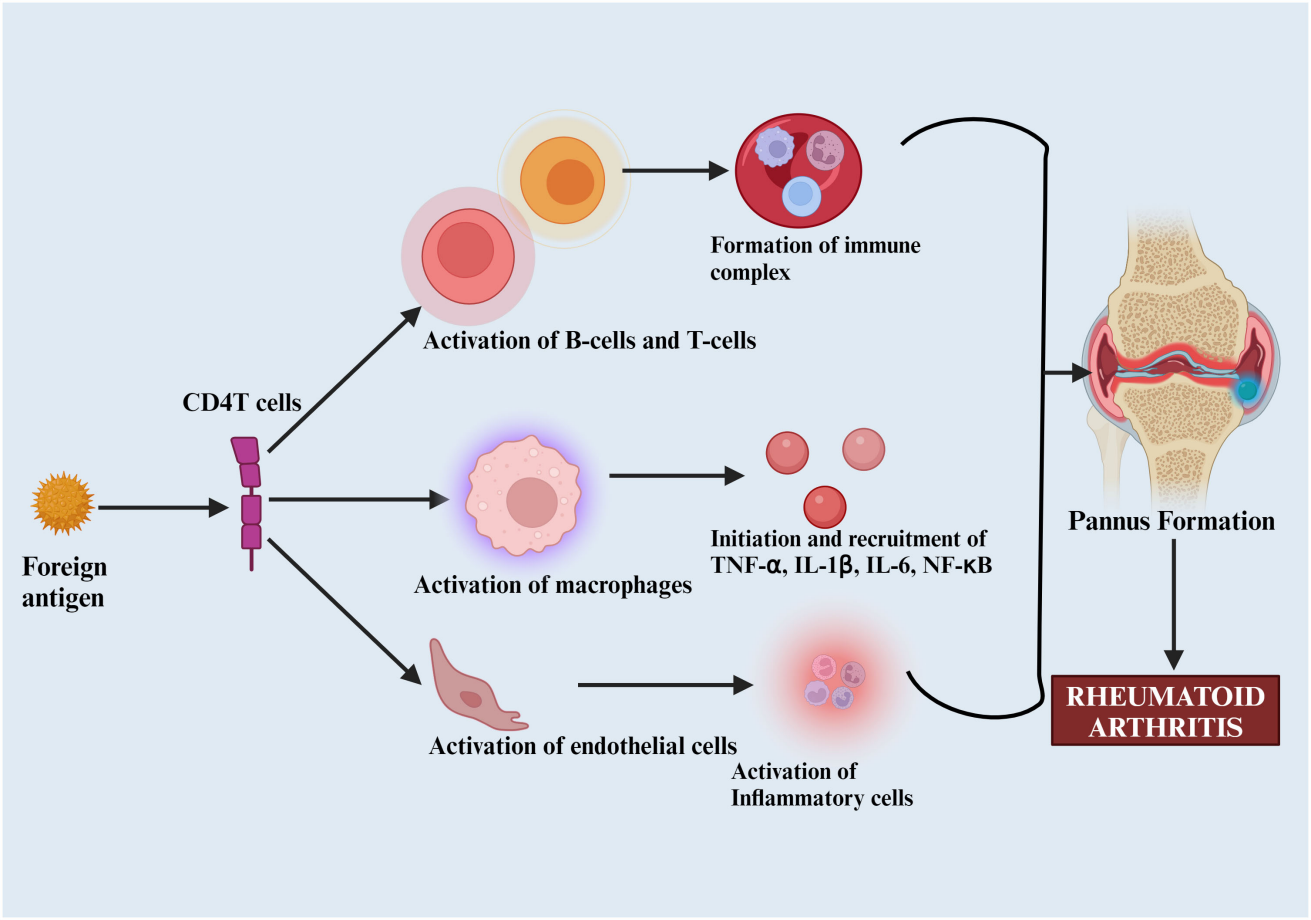
Pathophysiology of rheumatoid arthritis. Foreign antigens that activate B and T-cells, macrophages, and other inflammatory mediators (TNF-α, IL-1β, IL-6), leading to pannus formation and subsequent joint destruction.

The activation of major histocompatibility complex (MHC) Class II-dependent T-cells by citrullinated neoantigens promotes B-cells production of more ACPAs and the activation of inflammatory mediators like TNF-α, IL-1β, IL-6, and NF-κB, leading to pannus formation—a thickened synovial layer that invades and destroys cartilage and bone. This stage is typically characterized by a loss of immune tolerance (**Figure 3**) (50, 51).

Understanding the detailed pathophysiology of RA is critical for developing targeted therapies to manage and potentially alter disease progression. By elucidating these mechanisms, researchers can identify novel therapeutic targets and improve treatment strategies for RA.

## 3 Bee venom

BV is synthesized within the venom gland located in the bee’s abdominal cavity (52, 53). This complex mixture contains at least 18 active components, including active peptides, enzymes, amino acids, proteins, phospholipids, sugars, and other non-peptide elements (**Table 1**) (54, 55). Studies demonstrated the therapeutic potential of BV, highlighting its anti-atherosclerotic, antiarthritic, antimicrobial, and immunosuppressive activities, as well as its cytotoxic effects against cancer cells (56).

**Table 1:** Composition of Bee Venom.

Class of molecules	Components	% of dry BV
Proteins and peptides	Melittin	0-50
	Apamin	1-3
	Mast cell degranulation peptide	1-2
	Adolapin	1
Phospholipids	NA	5
Volatile components	NA	4-8
Sugar	Glucose and fructose	2
Amino acids	Aminobutyric acid, α-amino acids	2
Amines	Histamine	0.5-2
Enzymes	Phospholipase A2	10-12
	Hyaluronidase	1-3
	α-glucosidase	0.6-1

NA, not available.

BV has been used as a therapeutic agent in Korea and Eastern Asia since ancient times, even before the common era, where it was administered through injections or live bee stings to treat various conditions such as back pain, tumors, arthritis, and multiple sclerosis (44).

### 3.1 Basic components of bee venom

BV is primarily composed of melittin, a protein that constitutes approximately 55% to 60% of its dry weight. Another significant component is the mast cell degranulation peptide (MCD), also known as peptide 401, which accounts for about 2% to 3% of BV’s weight (56).

### 3.2 Physical properties of bee venom

BV is a translucent liquid with a pH range of 4.5 to 5.5, characterized by an unpleasant taste and odor. It is insoluble in ammonium sulfate and ethanol but is soluble in water (57).

## 4 Melittin

**Figure 4:**
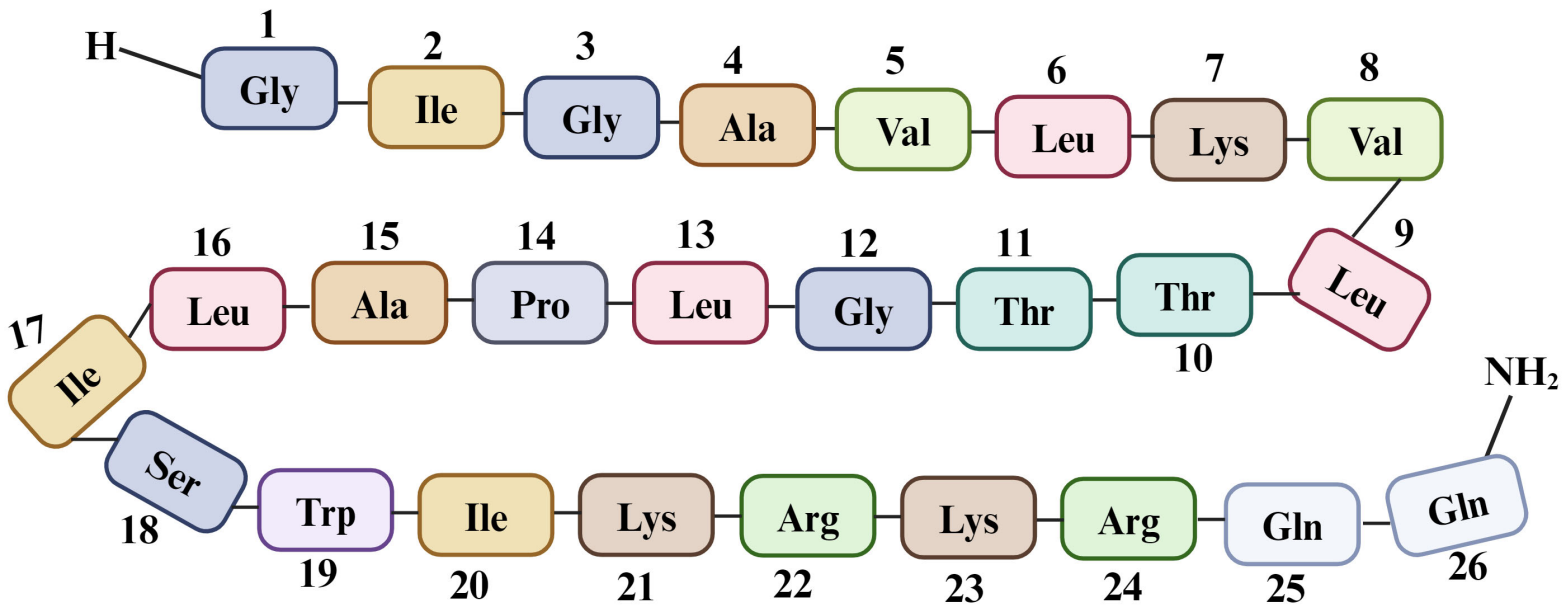
Sequence of 26 amino acid residues in melittin, a component of Bee venom. Melittin is synthesized as an inactive precursor within a bee’s venom gland known as “pre-pro melittin”, which consists of 70 amino acids. The active form of melittin exhibits polar characteristics, with the last six amino acids being hydrophilic and the first 20 amino acids (starting from the N-terminus) being hydrophobic (60). This arrangement of amino acids contributes to its unique three-dimensional structure and amphoteric nature, with a +6 charge at physiological pH (including a +2 charge at the C-terminal and a +4 charge at the N-terminal region). Due to this specific arrangement, melittin exhibits hemolytic activity (61).

Melittin is the key component of BV, comprising up to 52% of its dry mass. It is a basic peptide with a molecular weight of 2846.5 Da and the chemical formula C_131_ H_229_ N_39_ O_31_, consisting of 26 amino acids (58, 59) (**Figure 4**).

Melittin is responsible for multiple effects, including anti-inflammatory, antibacterial, and antiviral effects, in various cell types. As a basic peptide, melittin acts as a natural detergent with high membrane surface tension, disrupting the structure of the phospholipid bilayer by forming pores and aggregates in both synthetic and natural membranes (62). In addition to causing morphological changes in membranes, melittin stimulates various enzymes such as adenylate cyclase, protein kinase, G-protein, and phospholipase C and D (63). Structurally, melittin forms a bent rod shape with two α-helical segments connected by a coiled segment containing a proline residue, enabling it to penetrate cell membranes (64).

At low concentrations, melittin exists as a monomer that can lyse cells, but it forms tetramers at concentrations typically found in BV. The pain associated with melittin is due to the depolarization of nerve endings by these tetramers. When administered intravenously, the cytolytic peptides in melittin target all lipid membranes, leading to systemic toxicity (65).

### 4.1 Different sources of melittin

**Figure 5:**
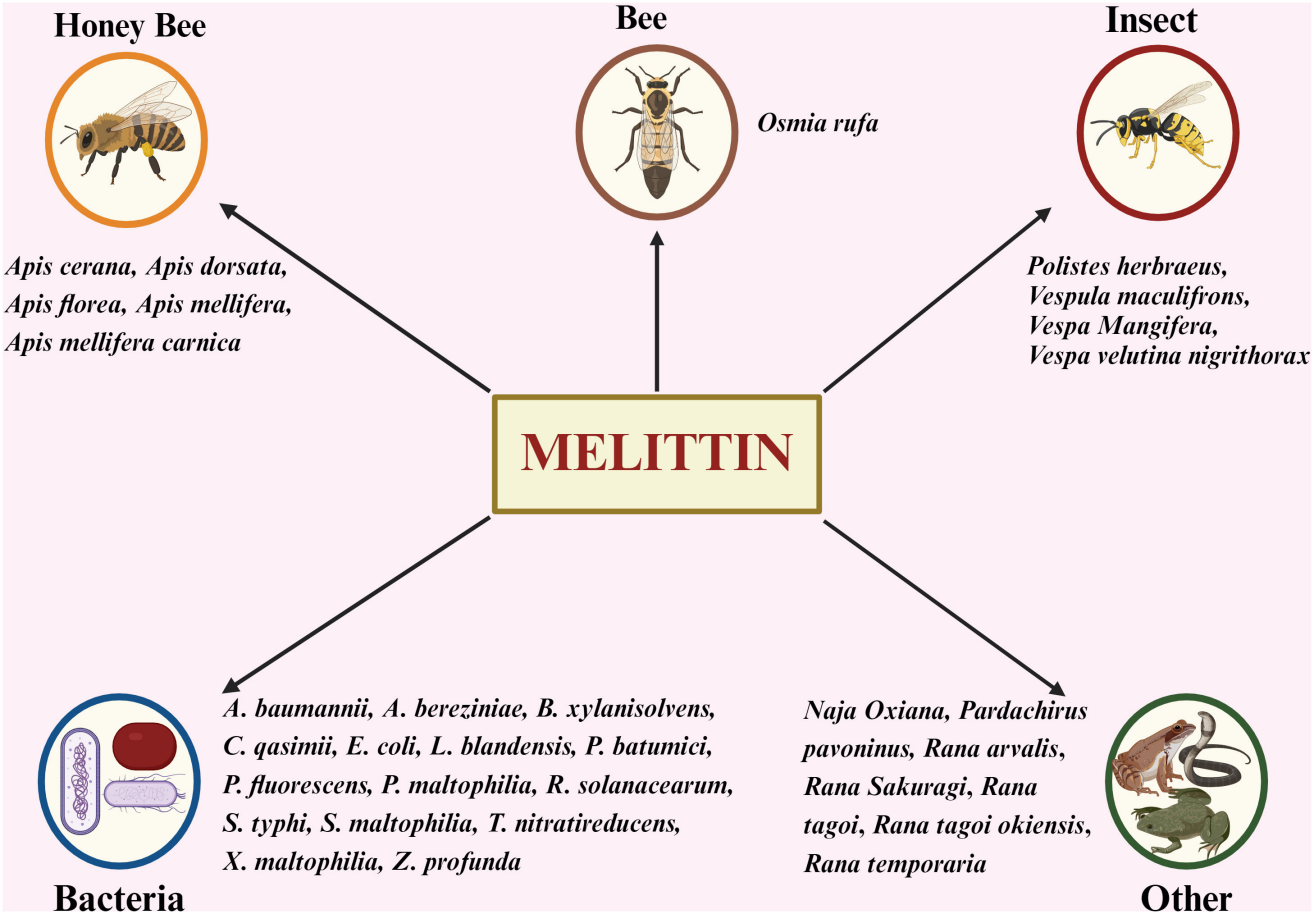
Exploring Diverse Origins: Sources of Melittin Beyond Honey Bees. *A. baumannii, Acinetobacter baumanni; A. bereziniae, Acinetobacter bereziniae; B. xylanisolvens, Bacteroides xylanisolvens; C. qasimii, Cyclobacterium qasimii; E coli, Escherichia coli; L. blandensis, Leeuwenhoekiella blandensis; P. batumici,Pseudomonas batumici; P. fluorescens, Pseudomonas fluorescens; P. maltophilia Pseudomonas maltophilia; R. solanacearum, Ralstonia solanacearum; S. typhi, Salmonella typhi; S. maltophilia, Stenotrophomonas maltophilia; T. nitratireducens, Thioalkalivibrio nitratireducens; X. maltophilia, Xanthomonas maltophilia; Z. profunda, Zunongwangia profunda*.

While BV from honeybees is the primary source of melittin, it is also found in various other species, including certain insects, bacteria, amphibians, and reptiles (**Figure 5**) (66).

### 4.2 Various pharmacological properties of melittin

Melittin exhibits various pharmacological properties, including anti-cancer, antimicrobial (anti-fungal, anti-protozoal, antiviral, antibacterial), antiarthritic, anti-diabetic, and anti-inflammatory activities (67–70). It disrupts cancer cell membranes through endocytosis, increasing membrane permeability and intracellular Ca²^+^, leading to apoptosis (67, 68).

Melittin has demonstrated antimicrobial activity against various pathogens (69, 71). For instance, melittin has shown efficacy in inhibiting *Mycoplasma gallisepticum* infection, particularly in plasmid isolates carrying the melittin gene (72). Additionally, the BV peptide lasioglossins, known for their DNA-binding capabilities and membrane interactions, exhibit even stronger antimicrobial effects (73). *In vivo* studies have shown that Melittin administration reduces TNF-α and IL-1β levels, as well as the infiltration of inflammatory cells in mouse skin following a *Dermatobacillus acne* injection (74, 75).

Melittin-loaded nanoparticles have demonstrated the ability to destroy the human immunodeficiency virus (HIV) without harming nearby healthy cells (59, 76). Melittin also interacts with the herpes simplex virus-1 (HSV-1), blocking its replication and reducing HIV-1 expression. Moreover, PLA2, another component of BV, may serve as an adjunctive antiviral agent in HIV treatment (77, 78).

Melittin has been demonstrated to enhance insulin synthesis by reducing the inflammatory response of the pancreatic islets (79). By depolarizing the membrane of pancreatic islets, melittin facilitates the opening of Ca²^+^ channels, allowing calcium ions to enter more easily and triggering B-cells to produce insulin (55, 80–82). The antiarthritic effect of BV was associated with a decrease in COX-2 and PLA_2_ expression, as well as a lower level of IL-1, IL-6, TNF-α, ROS, and NO. This occurs through a strong protein-protein interaction (PPI) that alters the actions of IKKβ and IKKα, restricting the release of IκBβ and IκBα, which are essential for the translocation of the p50 subunit of NF-κB. As a result, the binding ability of NF-κB to DNA is reduced, leading to decreased expression of proinflammatory genes (83, 84). The anti-inflammatory effects of melittin across various disease models at different therapeutic doses are summarized in **Table 2**.

**Table 2:** Therapeutic dose of melittin for anti-inflammatory effect for different disease models.

Disease model	Route of administration	Dose	Animal/cell line used	Inferences drawn	Reference
Atherosclerosis	NA	2 µg/ml (melittin)	Human vascular smooth muscle cells were stimulated with TNF-α. *In vitro*	↓ Expression of 1L-1β, TNF-α, & NF-кB	(85)
Atherosclerosis	NA	0.1-1µg/ml (melittin)	Macrophages derived from the human monocyte cell line THP-1 were cultured. *In vitro*	↓ IL-1β, TNF-α and NF-κB activation ↓ Phosphorylation of EGFR and ERK ↓ Expression of NF-κB in the nucleus	(86)
Atherosclerosis	IP	0.1mg/Kg (melittin)	C57/BL6 mice, male *In vivo*	↑ Serum HDL-C level ↓ IL-1β, TNF-α ↓ VCAM-1, ICAM-1 expression ↓ fibronectin, TGF-β1 expression	(86)
Neuro-inflammation	NA	0.5-2µg/ml (melittin)	BV2 microglia. *In vitro*	**↓** TNF-α, IL-1β, IL-6, PGE_2_ **┴** COX-2 **┴** NO, iNOS **↓** NF-кB activation Block IкBα degradation	(87)
Acne vulgaris	NA	0.1-1µg/ml (melittin)	Human THP-1 monocytic cell *In vitro*	**↓** IKK, NF-кB, p38 phosphorylation, swelling **┴** TNF-α, IL-1β, IL-8 and apoptosis **┴** cleavage of caspase 3, 8	(88)
Amyotrophic lateral sclerosis	SC	0.1µg/g 3 times a week (melittin)	Mouse (hSOD1G93A transgenic) *In vivo*	**↓** lba-1, CD14 (lungs) **↓** CD14 and COX-2 (spleen) **↑** pERK and Bcl2	(89)
Arthritis	NA	5, 10µg/ml (melittin)	RAW 264.7 mouse macrophases; Synoviocytes obtained from RA patient. *In vitro*	Melittin binds to IKKα & IKKβ **┴** TNF-α, IKKβ **┴** NF-кB activation and nuclear translocation of p50 subunit. **┴** LPS-induced COX-2, NO, iNOS, and PGE_2_	(90)
Arthritis	NA	0.5, 5µg/ml (melittin)	RAW 264, THP-1 human cell; Synoviocytes obtained from RA patient. *In vitro*	**┴** LPS and SNP-induced JNK activation **┴**TNF-α JNK inhibitor suppressed inhibitory effect of melittin on NF-κB activation **┴** LPS and SNP-induced NO, and PGE_2_ production	(91)
Arthritis	SC	20µg/kg, (melittin)	Wistar albino male rats *In vivo*	**↓** TNF-α, IL-6, IL-1β, TOL and OSI	(92)
Gouty arthritis	Intra-articular (Tibiotarsal)	0.5mg/kg (BV)	Adult male Sprague Dawley rats *In vivo*	**↓** TNF-α, IL-1β, IL-6, COX-2, iNOS and chemokines (MIP-1α, MIP-1β, MCP-1, GRO-α, MIP-2α)	(93)
Arthritis	SC	2, 4, 20 mg/kg, each day for a period of 15 days (BV)	Male Wistar albino rats *In vivo*	**┴** Enzymatic activity of PLA2 ┴ TNF-α, IL-1β, IL-6, TGF-β1	(94)
Peri arthritis hemeroscapularis	IM	0.0025mg/kg, once per day for 15 days (BV)	Human patients *In vivo*	↓ IL-1β, TNF-α, ↑ IL-10 improvement in motor function and mobility	(95)
Arthritis	IP	60mg/kg/day (BV)	Adult male Wistar rats, *In vivo*	↓ TNF-α, IL-1β, ↓ NF-кB signaling	(96)
Gastric ulceration	IP	2mg/kg for 7 days (BV)	Adult male Sprague-Dawley rats, *In vivo*	↓ Ulcer index ↓ cytokine levels ↓caspase-3 expression ↓ tissue eosinophil levels	(97)
Cholangiopathy	IP	0.1mg/kg (melittin)	C57BL/6 male mice *In vivo*	**↓** serum alkaline phosphatase, bilirubin ↓ TNF-α, IL-6, apoptosis ↓ NF-кB signaling, TGF-β1 expression ┴ liver fibrosis	(98)
Liver inflammation	NA	0.5-2µg/ml (melittin)	Mouse hepatocyte cell line AML12, *In vitro*	┴ apoptotic pathway ┴ activation of bcl-2, bax, NF-κB activation	(99)

TNF-α, tumor necrosis factor-α; IL, interleukin; NF-кB, nuclear factor kappa beta; EGFR, epidermal growth factor receptors; ERK, extracellular signal-regulated kinase; PGE_2_, prostaglandin E_2_; NO, nitric oxide; iNOS, inducible nitric oxide synthase; IKK, IκB kinase; CD, cluster of differentiation; COX, cyclooxygenase; JNK, c-Jun N-terminal kinases; GRO-α, growth-regulated gene-α; SNP, sodium nitroprusside; TGF-β1, Transforming growth factor beta 1; TOL, total oxidant level; OSI, oxidative stress index; LPS, lipopolysaccharide; VCAM, vascular cell adhesion molecule, ICAM, intercellular adhesion molecule; MIP, macrophage inflammatory protein; bcl-2, B-cell lymphoma protein-2; bax, B-cell lymphoma protein- associated X; NA, not available. Symbol: ↑ increased; **↓** suppression; **┴** inhibition.

### 4.3 mechanism of the antiarthritic effect of melittin

The therapeutic potential of BV, particularly its key component melittin, has been extensively studied in both *in vitro* and *in vivo* models, revealing multiple anti-inflammatory and immunomodulatory mechanisms.

Nam et al. (100) demonstrated that the aqueous phase of BV (molecular weight <20 kDa) contains components with significant anti-inflammatory properties. *In vitro* studies identified that various BV extracts, including n-hexane, ethyl acetate, and the aqueous phase derived from *Apis mellifera*, exhibit a potent inhibitory effect on COX-2 activity while sparing COX-1. The aqueous partition was further subdivided based on molecular weight into three fractions: BV1 (>20 kDa), BV2 (10–20 kDa), and BV3 (<10 kDa). Of these, BV2 and BV3 fractions showed the most pronounced inhibition of COX-2 activity while avoiding cytotoxicity, indicating the presence of specific moieties responsible for suppressing proinflammatory cytokine production (TNF-_α_ and IL-1β).

Further assessments in RAW 264.7 murine macrophage cell lines and synoviocytes from RA patients revealed similar anti-inflammatory effects of BV and melittin. Dose-dependent studies in macrophage models showed significant reductions in tissue inflammation, edema, and osteophyte formation with BV and melittin therapy at concentrations as low as 0.1–5 µg/kg, highlighting its therapeutic efficacy (90).

BV and melittin also effectively inhibit the production of nitric oxide (NO) and prostaglandin E2 (PGE2) in LPS-stimulated RAW 264.7 cells without cytotoxic effects. This inhibition parallels the effects observed with indomethacin, a conventional COX-2 inhibitor, and underscores BV’s capability to attenuate inflammatory mediators within synoviocytes of RA patients (84, 90).

**Figure 6:**
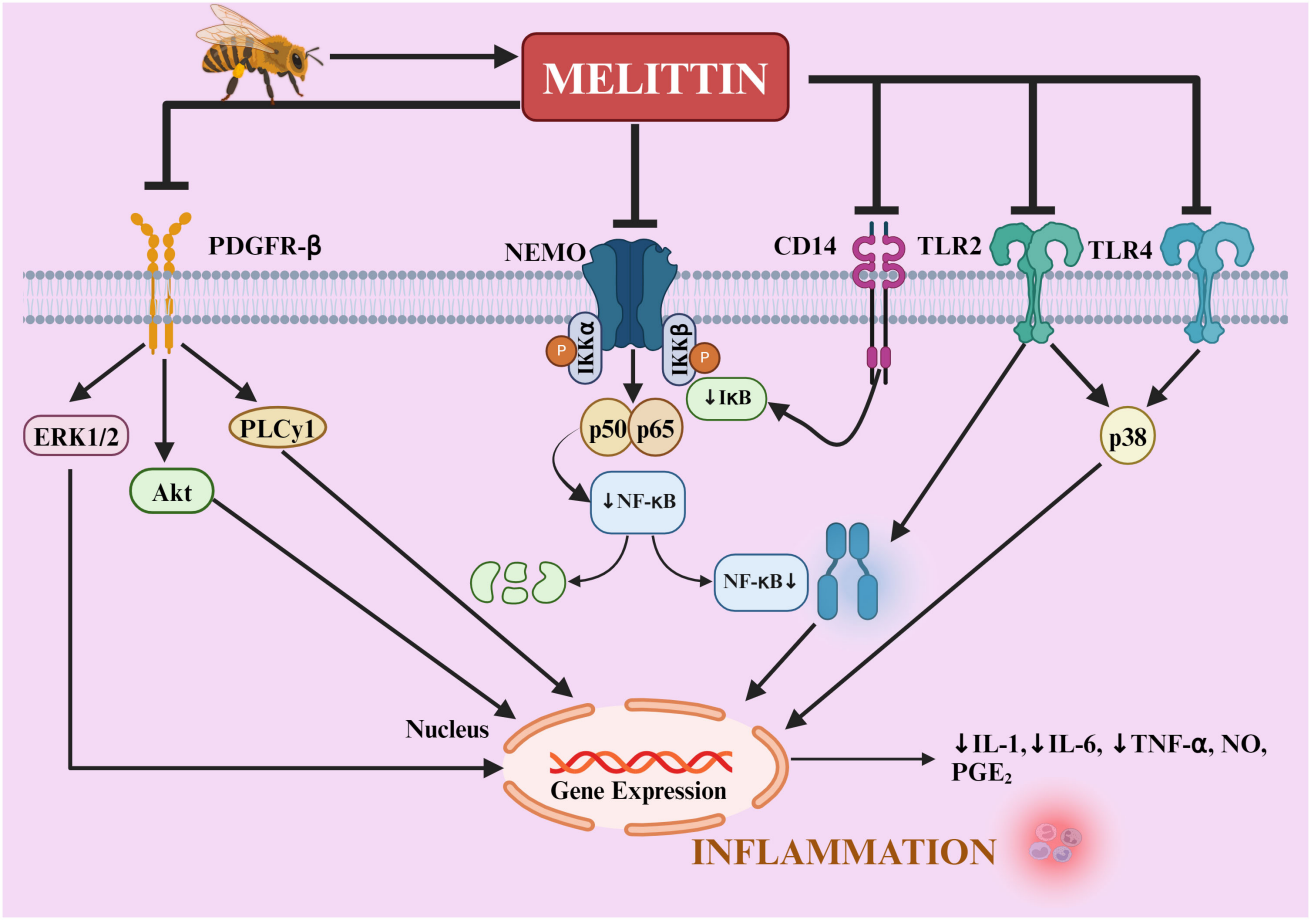
Mechanisms of melittin’s anti-inflammatory action in rheumatoid arthritis. melittin regulates TLR2, TLR4, CD14, NEMO, and PDGFRβ signaling pathways. Melittin blocks IKKs to prevent the release of IκB, thereby inducing NF-κB inactivation. It also decreases the activation of p38, ERK1/2, PLCγ1, and AKT, as well as the translocation of NF-κB into the nucleus, thereby reducing the inflammatory mediators (IL-1β, IL-6, TNF-α, NO, PGE2, ROS) in the liver, aorta, joints, skin, and neural tissue. NF-κB, nuclear factor-κB; IκB, inhibitor of NF-κB; IKK, IκB kinase; NEMO, NF-κB essential modulator; TNF-α, tumor necrosis factor; IL, interleukin; NO, nitric oxide; PGE2, prostaglandin E2; ERK1/2, extracellular signal-regulated protein kinases 1 and 2; CD, cluster of differentiation; TLR, toll-like receptor; PDGFR-β, platelet-derived growth factor receptor-β; PLCγ1, phospholipase Cγ1; Akt, protein kinase B.

Melittin’s mechanism of action extends to the suppression of NF-κβ activity, achieved by inhibiting IκB kinases (IKK_α_ and IKK_β_), reducing Iκβ phosphorylation, and preventing p50 translocation into the nucleus (84). These molecular actions culminate in decreased expression of proinflammatory genes, such as those encoding COX-2 and inducible nitric oxide synthase (iNOS) (**Figure 6**). Surface plasmon resonance analyses provide direct evidence of melittin’s interaction with these critical upstream signaling molecules, with dissociation constants of 4.6 × 10^-6^ M (IKK_α_), 1.34 × 10^-9^ M (IKK_β_), and 1.01 × 10^-9^ M (p50), highlighting its specificity and potency (90, 91). This strong PPI alters the actions of IKKβ and IKKα, restricting the release of IκBβ and IκBα, which are essential for p50 translocation, thereby reducing NF-κB’s ability to bind DNA.

The therapeutic effect of melittin has been further corroborated by studies showing its ability to reduce TNF-α, NO, and ROS levels—key mediators in inflammatory arthritis. These effects collectively contribute to its capacity to alleviate joint destruction and systemic inflammation (92–94, 101–103).

The potential protective mechanism of melittin against the inflammatory response is illustrated in **Figure 6**.

The inhibitory effects of melittin and BV on c-Jun N-terminal kinases (JNK) signaling were observed at concentrations of 5 µg/mL and within a range of 0.1–5 µg/mL, respectively. These effects were further validated by the use of SP600215, a specific JNK signal inhibitor, which suppressed the anti-inflammatory actions of melittin and BV. While other signaling pathways, such as p38 MAP kinase and ERK, may also be modulated depending on the cell type and stimulus, the specific inhibition of JNK signals appears to be a pivotal mechanism in reducing NF-κB activity and inflammatory mediator production. These findings demonstrate that melittin and BV exert their anti-inflammatory effects through a dual mechanism, targeting both NF-κB and JNK pathways. This dual inhibition contributes to the suppression of pro-inflammatory mediators, including NO and PGE2, underscoring the therapeutic potential of melittin and BV in managing RA and other inflammatory conditions (90, 91).

Synthetic melittin has been shown to bind to PLA2 and inhibit the enzymatic activity of secretory PLA2 (sPLA2) in synovial fluid taken from RA patients. This suggests that BV or its components may have a therapeutic role in disrupting key inflammatory enzymes, making the downregulation of genes that prevent inflammation crucial for its therapeutic efficacy (103, 104).

Yin et al. (105) conducted microarray analyses to investigate the global gene expression patterns in human chondrocyte-like cells exposed to BV. Their study revealed significant downregulation of key genes associated with inflammation, including the IL-6, matrix metalloproteinase-15 (MMP-15), caspase-6 and TNF-α ligand. These findings highlight BV’s ability to modulate inflammatory pathways at the transcriptional level, providing molecular evidence for its anti-inflammatory properties. The inhibition of these genes reflects BV’s potential to regulate critical processes involved in joint inflammation and destruction, further supporting its therapeutic utility in rheumatoid arthritis.

This transcriptional regulation complements the previously described suppression of pro-inflammatory mediators, such as NO, PGE2, and ROS, and the inhibition of NF-κB and JNK signaling pathways. Together, these molecular mechanisms underline melittin’s role in mitigating the systemic and localized inflammatory responses characteristic of RA. Moreover, the modulation of IL-6, MMP-15, and TNF-α ligands by BV adds another dimension to its therapeutic promise, targeting not only downstream inflammatory mediators but also upstream regulators of inflammation.

Additionally, BV-induced apoptosis in rheumatoid synovial fibroblasts has been shown to play a critical role in mitigating synovial hyperplasia, a hallmark of RA. By activating caspase-3 and modulating the balance between BAX and BCL-2 expression, BV promotes apoptosis and inhibits the proliferation of synovial cells, thereby alleviating the pathological effects of RA on joint tissues. This apoptotic mechanism not only reduces synovial cell density but also addresses one of the core drivers of joint destruction and chronic inflammation in RA (106). The dual action of melittin in targeting both inflammatory mediators and hyperplastic synovial cells highlights its potential as a multifaceted therapeutic agent for managing RA.

The collective evidence from these studies establishes melittin and BV as promising candidates for the development of novel RA treatments, particularly in cases where conventional therapies fail or cause significant side effects.

## 5 Melittin Formulations

### 5.1 Melittin transdermal delivery via polymeric microneedle for treatment of RA

Transdermal drug delivery systems offer a promising alternative for the treatment of RA, aiming to bypass the limitations associated with oral and injectable administration. Among these, melittin-loaded hyaluronic acid (HA) polymeric microneedles (Mel-HA-MN) represent a novel approach to enhance the localized and efficient delivery of melittin, a bioactive peptide known for its anti-inflammatory and immunomodulatory properties (107). Importantly, the microneedle delivery system significantly modulated the immune response in rodent models of adjuvant-induced arthritis (AIA). These Mel-HA-MN have shown potential in elevating regulatory CD4+ T cells and reducing levels of proinflammatory cytokines (TNF-α and IL-17), which may be linked to the modulation of T cells and cytokine activity (107, 108).

Mechanical characterization of the microneedles revealed a slight reduction in stress capacity upon melittin loading, with HA microneedles decreasing from 58 MPa to 38 MPa and Mel-HA-MN from 30 MPa to 27 MPa. Despite this reduction, the microneedles retained sufficient strength for effective skin penetration. The therapeutic efficacy of Mel-HA-MN was evaluated using the rat adjuvant-induced arthritis (AIA) model, where the microneedles’ penetration ability was tested by applying the patch on the rat’s abdominal skin (107).

Results demonstrated that Mel-HA-MN effectively penetrated the skin to a depth of approximately 200 µm. This depth ensures adequate delivery of melittin into the dermal layer, where it exerts its therapeutic effects. The therapeutic potential of Mel-HA-MN was further validated in AIA rodent models, where repeated applications of the microneedle patch successfully reduced paw swelling, maintained body weight and preserved cartilage integrity. By the end of the treatment, the paw thickness of treated animals decreased to less than half of that observed in the untreated control group, and clinical scores were significantly improved, matching the efficacy of subcutaneous melittin administration (107).

A key innovation in this approach is the incorporation of methacrylate-modified HA (MeHA), enabling sustained release of melittin. This modification prolonged therapeutic action and reduced administration frequency, highlighting its potential for long-term management of RA. The ability to tune drug release profiles adds an element of flexibility to this delivery method, paving the way for personalized treatment regimens.

The results of these studies emphasize the clinical relevance of Mel-HA-MN as a targeted, non-invasive treatment strategy for RA (107). By overcoming the challenges of direct administration, such as systemic toxicity and patient compliance issues (109), this technology holds significant promise for improving RA management.

### 5.2 Nanoemulsions of bee venom

Nanoemulsions containing BV have been developed and studied for their potential to reduce inflammation in animal models of RA. Research suggests that melittin’s antiarthritic effects can be enhanced through water-in-oil (W/O) nanoemulsion formulations containing BV (BV-NEs), which incorporate a surfactant mixture and an oil phase. These formulations have been tested on collagen-induced arthritis (CIA) in male Wistar rats (110).

A skin permeation test was conducted by applying BV-NE for up to 12 hours, revealing increased permeability with higher BV content in the formulations. Studies showed a significant decrease in serum levels of TNF-α and IL-17 from day 14 of the treatment, with further reductions observed on day 21. This demonstrates that nanoemulsions loaded with BV effectively reduce serum levels of IL-17 and TNF-α, indicating a modulation of both adaptive and innate immune responses following two weeks of topical treatment (110).

By encapsulating melittin in a nanoemulsion, BV-NEs improve the bioavailability and stability of the peptide while allowing for a controlled and sustained release. This not only enhances therapeutic efficacy but also reduces the risk of systemic side effects, making BV-NEs a safer alternative to traditional systemic therapies. In conclusion, nanoemulsions of bee venom represent an innovative and effective delivery system for the treatment of RA.

### 5.3 Incorporation of bee venom gel via micro-needling

In this study, BV gel was combined with a transdermal drug delivery system utilizing micro-needling to provide a precise and effective treatment option for RA. This approach leverages the ability of microneedles to create microchannels in the skin, facilitating the penetration and localized action of BV’s active components. The experiment involved inducing acute gouty inflammation in rats using monosodium urate crystal (MUC) and acute inflammation in mice using LPS. The anti-inflammatory effects were assessed by evaluating the permeability of the prepared microneedle gel.

A direct correlation was observed between the percutaneous absorption of the prepared microneedle gel and the reduction in NO levels in both the MUC and LPS-induced inflammation models.

The BV gel was prepared using carboxymethylcellulose sodium, a colorless, odorless, translucent material, as the gel matrix, along with antioxidants such as thiourea, sodium thiosulfate, glucose, and stabilizer. In patch skin tests, it was observed that melittin successfully penetrates the stratum corneum with the assistance of microneedles. Notably, the stability of melittin within the gel preparation remained intact after the addition of 0.1% stabilizer, maintaining its stability for up to 6 months. The study found that applying a force of 10 N with a 750 µm microneedle for 3 minutes produced the greatest anti-inflammatory effect (111).

The approach provides a localized and minimally invasive alternative to conventional treatments, ensuring targeted delivery while minimizing systemic exposure and associated risks.

## 6 Challenges with melittin as a therapeutic agent

Safety concerns have been one of the primary challenges associated with the therapeutic use of BV, particularly melittin. Despite numerous reports of adverse reactions, no comprehensive systematic analyses on safety in clinical practice have been conducted to date. The key issue with melittin is its potential to provoke the breakdown of intracellular and plasma membranes, as well as its ability to act as an allergen, inducing IgE-mediated responses that can lead to allergic reactions in several patients (112).

Melittin exhibits hemolytic activity and possesses cytotoxic and genotoxic effects (113, 114). It can disrupt lipid bilayers by acting as a natural surfactant, interacting with phospholipids, and integrating into red blood cell (RBC) membranes. This interaction with plasma membranes results in various effects, including the disruption of phospholipid packaging in the lipid bilayer, the formation of channels and pores, the aggregation of membrane proteins, and the induction of spontaneous cell lysis. When administered in high doses, melittin can cause itching, local reactions, and pain. However, at lower concentrations, it may exhibit beneficial anti-inflammatory effects, primarily due to the inhibition of PLA2 (115). Nonetheless, melittin’s administration appears to have cytotoxic effects on normal human cells, potentially raising mRNA levels of oxidative stress and apoptosis-related genes (58).

To address these challenges, several strategies have been explored to minimize the adverse effects of melittin while retaining its therapeutic potential. Asthana et al. (116) demonstrated that melittin’s hemolytic activity can be significantly reduced by substituting alanine in the leucine zipper motif. Additionally, Rayalin et al. (117) found that a melittin fusion protein with glutathione S-transferase exhibits low toxicity and retains anti-inflammatory properties.

The development of delivery techniques utilizing nanocarriers has shown promise in safely delivering melittin to specific lesions while minimizing harm to non-targeted cells. For example, Gui and colleagues (118) designed a polyelectrolyte-based nano-complex system using flash nanocomplexation technology. This system forms strong interactions between negatively charged dextran sulfate and positively charged melittin, resulting in decreased acute toxicity and enhanced pathological indicators, thereby increasing melittin’s therapeutic potential (118).

Investigating the co-treatment of PLA2 and melittin could be worthwhile, as they may complement each other well. Additionally, combining melittin with other natural products, such as curcumin or resveratrol, may provide synergistic effects and further reduce toxicity (119–121).

## 7 Conclusion and future aspect

BV is a complex biological mixture containing various bioactive components, such as peptides (notably melittin and PLA2), phospholipids, proteins, amino acids, enzymes, carbohydrates, minerals, and small amounts of volatile components. Historically used in traditional Chinese medicine to manage a range of conditions, like pain, arthritis, rheumatism, and skin diseases. BV and its components continue to gain attention for their therapeutic potential. Among these components, melittin has emerged as a particularly potent agent with significant anti-inflammatory effects. Its mechanism includes the inhibition of NF-κB activity by preventing IκB phosphorylation, which ultimately suppresses the expression of proinflammatory genes central to RA pathophysiology.

While existing RA treatments, such as NSAIDs, DMARDs, and biologics, offer varying degrees of symptom control, they often have limitations, including side effects, high costs, and limited efficacy for some patients. In this context, melittin offers a novel and promising approach. However, its cytotoxic potential presents challenges requiring further research to enhance its safety profile. The review highlights recent advancements in the formulation of melittin, such as transdermal patches, microneedle-delivered gels, and nanoemulsion-based topical applications, which may enhance its therapeutic delivery and minimize adverse effects.

Future research should focus on a deeper understanding of the molecular and cellular mechanisms underlying melittin’s antiarthritic activity. Continued work is essential to refine its delivery systems and assess its efficacy and safety across broader animal models, especially primates, to approximate human clinical conditions. Sustainable and standardized methods of BV extraction will also be essential to ensure consistent and reliable production.

Future studies should prioritize to investigate the key components of BV, particularly melittin and PLA2, to gain a deeper understanding of their physicochemical properties and enhance their therapeutic potential. Additionally, exploring complementary or alternative compounds—such as apamin, mast cell degranulating peptide, curcumin, and resveratrol—either individually or in combination with melittin could mitigate cytotoxicity while enhancing therapeutic efficacy. By addressing these challenges, optimizing combination therapies, and refining delivery system, melittin and other BV components may ultimately offer safer, more effective and accessible options for RA management and other inflammatory conditions. These efforts have the potential to expand treatment strategies and improve patient outcomes, presenting BV as a versatile foundation for future therapeutic advancements.
